# Postmating isolation and evolutionary relationships among *Fejervarya* species from Lesser Sunda, Indonesia and other Asian countries revealed by crossing experiments and mtDNA Cytb sequence analyses

**DOI:** 10.1002/ece3.9436

**Published:** 2022-10-22

**Authors:** Mahmudul Hasan, Nia Kurniawan, Aris Soewondo, Wilmientje Marlene Mesang Nalley, Masafumi Matsui, Takeshi Igawa, Masayuki Sumida

**Affiliations:** ^1^ Department of Fisheries Bangamata Sheikh Fojilatunnesa Mujib Science and Technology University Jamalpur Bangladesh; ^2^ Amphibian Research Center Hiroshima University Higashihiroshima Japan; ^3^ Department of Biology, Faculty of Mathematics and Natural Sciences Brawijaya University Malang East Java Indonesia; ^4^ Faculty of Animal Science University of Nusa Cendana Kupang Indonesia; ^5^ Graduate School of Human and Environmental Studies Kyoto University Kyoto Japan; ^6^ Present address: 1‐6‐15 Ushitaasahi Hiroshima Japan

**Keywords:** *Fejervarya*, mtDNA, reproductive/Postmating isolation, species

## Abstract

To evaluate the degree of postmating isolation and the evolutionary relationships among frog species in the genus *Fejervarya* from Indonesia (Lesser Sunda), Bangladesh, China, and Japan, crossing experiments and molecular phylogenetic analyses were carried out. Crossing experiments revealed that reciprocal hybrids among *F. iskandari*, *F. verruculosa*, and *F*. sp. large type and between *F. multistriata* and *F. kawamurai* are viable through metamorphosis, while those between the *F. iskandari* group and *F. limnocharis* group were completely or partially inviable at the tadpole stage and those between Southeast Asian and South Asian *Fejervarya* groups were completely inviable at the embryonic stage. The mature reciprocal hybrids between *F. iskandari* and *F. verruculosa* from Lesser Sunda, Indonesia, showed some degree of abnormality in spermatogenesis. In phylogenetic analyses based on mtDNA *Cytb* sequences, *F. iskandari* formed a sister clade with *F. verruculosa* from Lesser Sunda, Indonesia, with 8.1% sequence divergence. *F. multistriata* from China formed a clade with populations of *F. limnocharis* in Thailand, Malaysia, and Indonesia (topotype), and these taxa showed sister relationships to *F. kawamurai* from Japan with 8.9% sequence divergence. *Fejervarya* sp. small type from Bangladesh formed a clade with the other South Asian members of the *Fejervarya* group and formed a sister clade with the Southeast Asian *Fejervarya* group, with 23.1% sequence divergence in the *Cytb* gene. These results showed that the degree of postmating isolation reflects molecular phylogenetic relationships and that *F. iskandari* and *F. verruculosa* from Indonesia (Lesser Sunda) are reproductively isolated by abnormalities in spermatogenesis and show genetic differentiation.

## Graphical abstract

In order to interpret the degree of postmating isolation and the evolutionary relationships among *Fejervarya* species from Indonesia and other Asian countries, crossing and molecular experiments were done. This study showed that the degree of postmating isolation reflects the molecular phylogenetic relationships, and that the two species, *F. iskandari* and *F. verruculosa* from Indonesia (Lesser Sunda) are reproductively isolated by abnormal spermatogenesis, and genetically deviated.

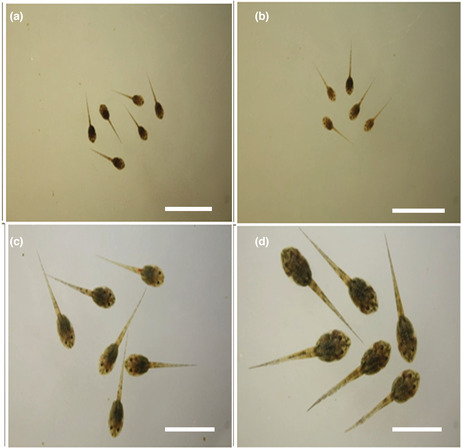

## 1 INTRODUCTION

A species is an indispensable entity in biological science (Claridge et al., 1997; Ereshefsky, 1992; Mayr, 1982), and recent approaches for species identification are debated among scientists and yield conflicting results for targeted taxa; thus, the perception of what constitutes a species continues to be a challenging dilemma (de Queiroz, 1998; Dobzhansky, 1976). Although Mayden (1997, 1999) drafted around 24 titled species concepts, the biological species concept remains the most central definition of the qualities of distinct species. If two taxa are reproductively isolated and cannot interbreed, they should be recognized as distinct species. It is possible that species are first isolated from each other, either completely or incompletely, by gametic isolation. When gametic isolation is incomplete, species are completely or incompletely isolated by hybrid inviability, and when hybrid inviability is incomplete, species are completely isolated by hybrid sterility (Sumida et al., 2003).

The most widely distributed frogs in Asia are *Fejervarya limnocharis* (Annandale, 1918). Recently, the species was transferred from the genus *Rana* to *Fejervarya* (Dubois & Ohler, 2000). This species is widely distributed in South to Southeast Asia, including many islands in Indonesia and Malaysia, northern, central, southern, and southwestern China, and Western Japan. A lack of diagnostic morphological characters makes it difficult for researchers to clearly separate these frogs, which are collectively referred to as the *Fejervarya limnocharis* complex. Many scientists have focused on the systematics of the *Fejervarya limnocharis* species complex. This species was first described in Java, Indonesia (Gravenhorst, 1829; Wiegmann, 1834). To date, 14 species have been listed in the genus *Fejervarya* (Frost, 2021), and several species have been described in this species complex. Additional analytical work and more extensive studies are needed to delimit all distinct species within the *F*. *limnocharis* complex along with their geographic ranges. Djong et al. (2007) argued that the *F*. *limnocharis* complex can be divided into two subgroups (i.e., the *F*. *limnocharis* group and *F*. *iskandari* group). In this classification, the *F*. *limnocharis* group includes the topotypic *F*. *limnocharis* (Java specimen, Indonesia) and populations in Malaysia and Japan, whereas the *F*. *iskandari* group consists of *F*. *iskandari* and populations from Thailand and Bangladesh.

Analyzing and quantifying biological heterogeneity is a substantial scientific endeavor (Rivera‐Correa et al., 2021). Only a tiny portion of the species on Earth has been explored, described, and identified, and we are far from generating an all‐inclusive inventory of the biosphere (Moura & Jetz, 2021; Wheeler et al., 2012). Importantly, *Fejervarya* specimens from Kupang, Ende and Maumere, Lesser Sunda have not been explored by artificial breeding to clearly determine whether the population belongs to the *F*. *iskandari* group or not. Therefore, in the current study, we examined specimens from the Lesser Sunda Islands to determine their phylogenetic affinities and to determine levels of reproductive isolation (if any) between the population and other known species from mainland Asia.

In the present study, a crossing experiment with six species was performed to clarify the reproductive isolating mechanisms. In addition, mtDNA gene sequence analyses of 27 frogs belonging to the genus *Fejervarya* from Indonesia, Bangladesh, Japan, and China were performed to determine the evolutionary relationships and levels of divergence within the *F*. *limnocharis* complex.

## 2 MATERIALS AND METHODS

### 2.1 Crossing experiments

**FIGURE 1:**
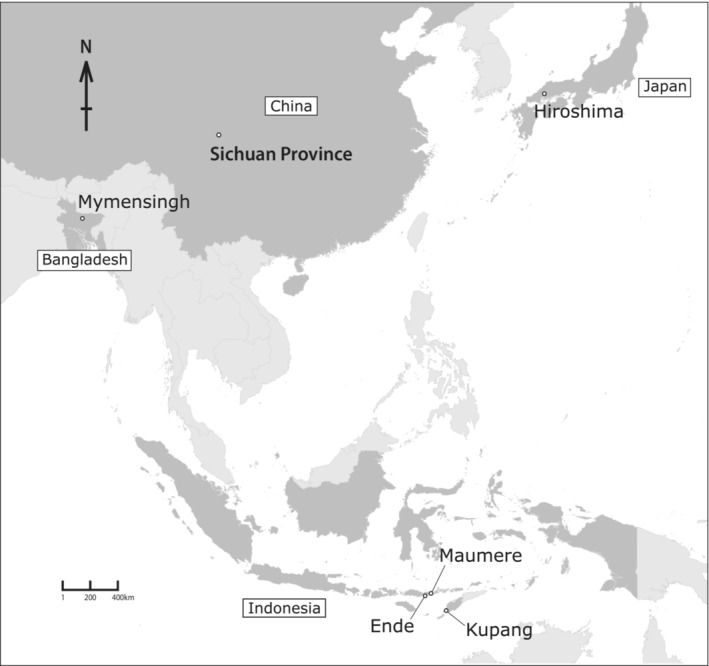
Map showing the localities of *Fejervarya* species used in the present study.

**FIGURE 2:**
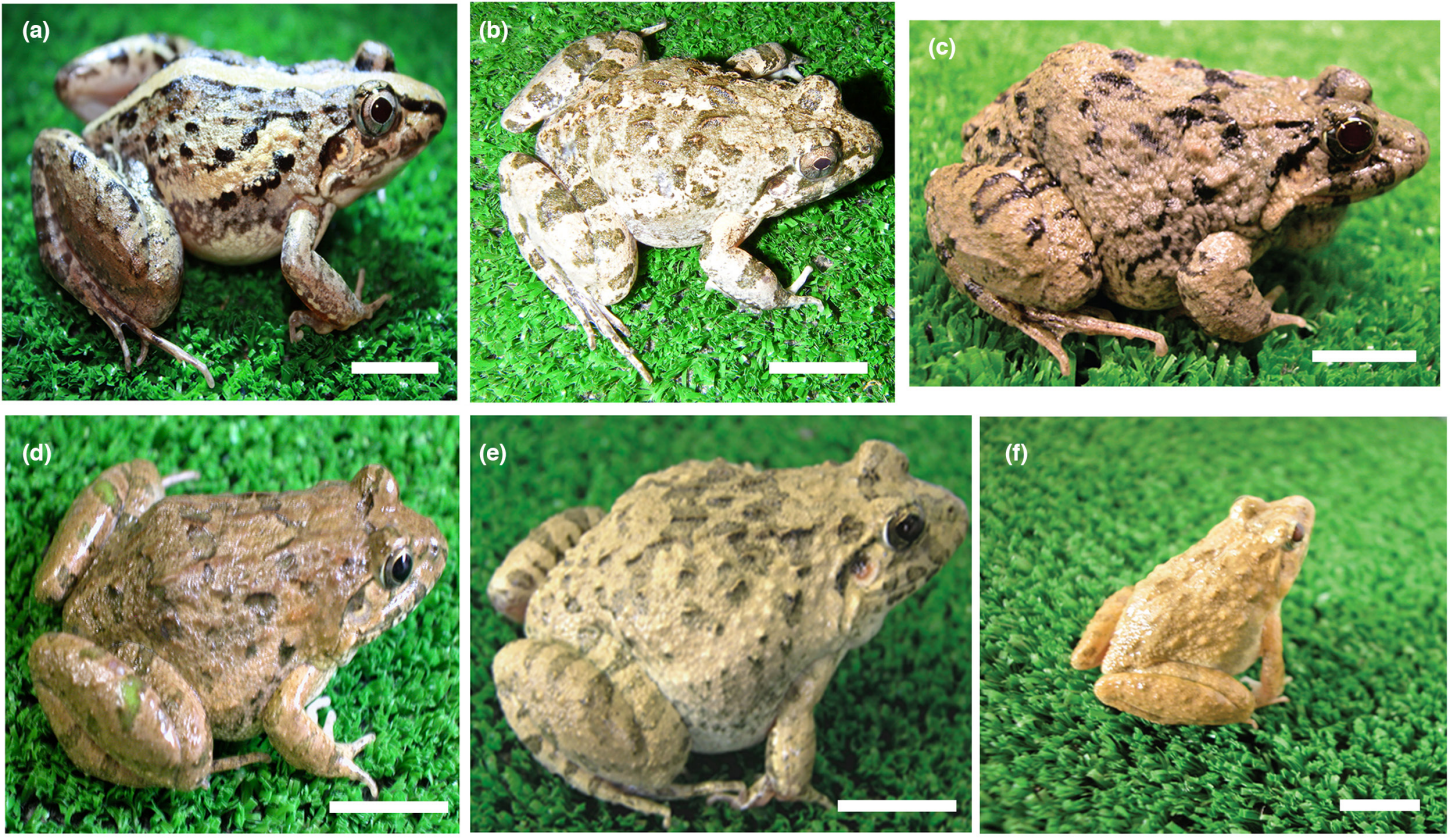
Six *Fejervarya* species used for crossing experiments. (a) *F. iskandari* (Indonesia), (b) *F. verruculosa* (Indonesia), (c) *F*. sp. large type (Bangladesh), (d) *F. multistriata* (China), (e) *F. kawamurai* (Japan), (f) *F*. sp. small type (Bangladesh). Scale bar = 10 mm.

Crossing experiments were performed by artificial insemination (Kawamura et al., 1980) during the breeding season (i.e., on August 20, 2010 and May 10, 2012) using 15 frogs (8 females and 7 males) belonging to six species of the genus *Fejervarya* from Indonesia, Bangladesh, Japan, and China (Table 1, Figures 1, 2). Sperm suspensions were prepared by crushing a single testis removed from each male in 2–3 ml of distilled water. Ovulation was expedited by the injection of bullfrog pituitary extract into the body cavity, and the released eggs were stripped from the females and placed on glass slides. After sperm motility was visually confirmed under a microscope, eggs were inseminated with the sperm suspension, transferred to glass Petri dishes containing 400–450 ml of tap water, and then observed to confirm typical progress. The resulting tadpoles were fed boiled spinach and metamorphosed frogs were fed crickets. Viability was calculated as the proportion of eggs showing ordinary development at each of the subsequent developmental stages: normal cleavage, tail‐bud embryo, hatched tadpole, feeding tadpole, 23‐ to 30‐day‐old tadpole, and metamorphosed frog.

**TABLE 1:** Frogs used for crossing experiments.

Species	Country	Location	No. of frogs			Abbreviation
			Total	♀	♂	
*Fejervarya verruculosa*	Indonesia	Ende, Lesser Sunda	1	1	0	Fver(E)
	Indonesia	Maumere, Lesser Sunda	2	1	1	Fver(M)
*Fejervarya iskandari*	Indonesia	Ende, Lesser Sunda	3	2	1	Fisk(E)
	Indonesia	Kupang, Lesser Sunda	1	0	1	Fisk(K)
*Fejervarya* sp. large type	Bangladesh	Mymensingh	2	1	1	Fsp.L
*Fejervarya* sp. small type	Bangladesh	Mymensingh	1	0	1	Fsp.S
*Fejervarya kawamurai*	Japan	Hiroshima	4	2	2	Fkaw
*Fejervarya multistriata*	China	Sichuan province	1	1	0	Fmul
Total			15	8	7	

### 2.2 Histological and spermatogenesis observations

Testes of the mature hybrids and control frogs were used for histological and spermatogenesis examinations. For each individual, one testis was fixed in Navashin's solution, sectioned at 10 μm, and stained with Heidenhain's iron hematoxylin for histological analyses. The other testis was used for chromosome preparations. Meiotic chromosomes were prepared according to the procedure described by Schmid et al. (1979) with minor alterations. The chromosomes were stained with a 2% Giemsa solution for 5 min. The chromosome analysis was performed using only diploid cells at the diakinesis and metaphase stages of the first reduction division, as bivalent and univalent chromosomes could be conveniently differentiated from each other. Bivalent chromosomes were similar to normal chromosomes, with a thick, symmetrical form, whereas univalent chromosomes were indistinguishable from abnormal chromosomes, which were asymmetric and lean (Kawamura et al., 1980, 1981; Kuramoto, 1983; Sumida et al., 2003).

### 2.3 mtDNA sequencing and data analyses

A total of 27 frogs belonging to the genus *Fejervarya* were used for genetic research. Genomic DNA for PCR was extracted from clipped toes using the DNAeasy Tissue Kit (Qiagen, Valencia, CA, USA) following the manufacturer's instructions. The extracted DNA solutions were used to amplify a partial region of the *Cytb* gene corresponding to nucleotide positions 14,396–15,063 in the *Hoplobatrachus tigerinus* complete mtDNA sequence (accession no. AP011543, Alam et al., 2010). PCR amplification and sequencing were performed using the primers Fow‐1‐1 and Rev‐1 (Hasan et al., 2012) to obtain a fragment of ca. 667 bp. The resultant nucleotide sequences were aligned using the ClustalW program (Thompson et al., 1994). A phylogeny was constructed using the maximum likelihood (ML) method implemented in Treefinder (Jobb, 2008), and branch support was evaluated by a bootstrap analysis with 100 replicates. The resultant sequence data were deposited in the DDBJ database under accession nos. LC706527–LC706542. Additional *Cytb* data were obtained from GenBank for tree construction.

## 3 RESULTS

### 3.1 Crossing experiments

**FIGURE 3:**
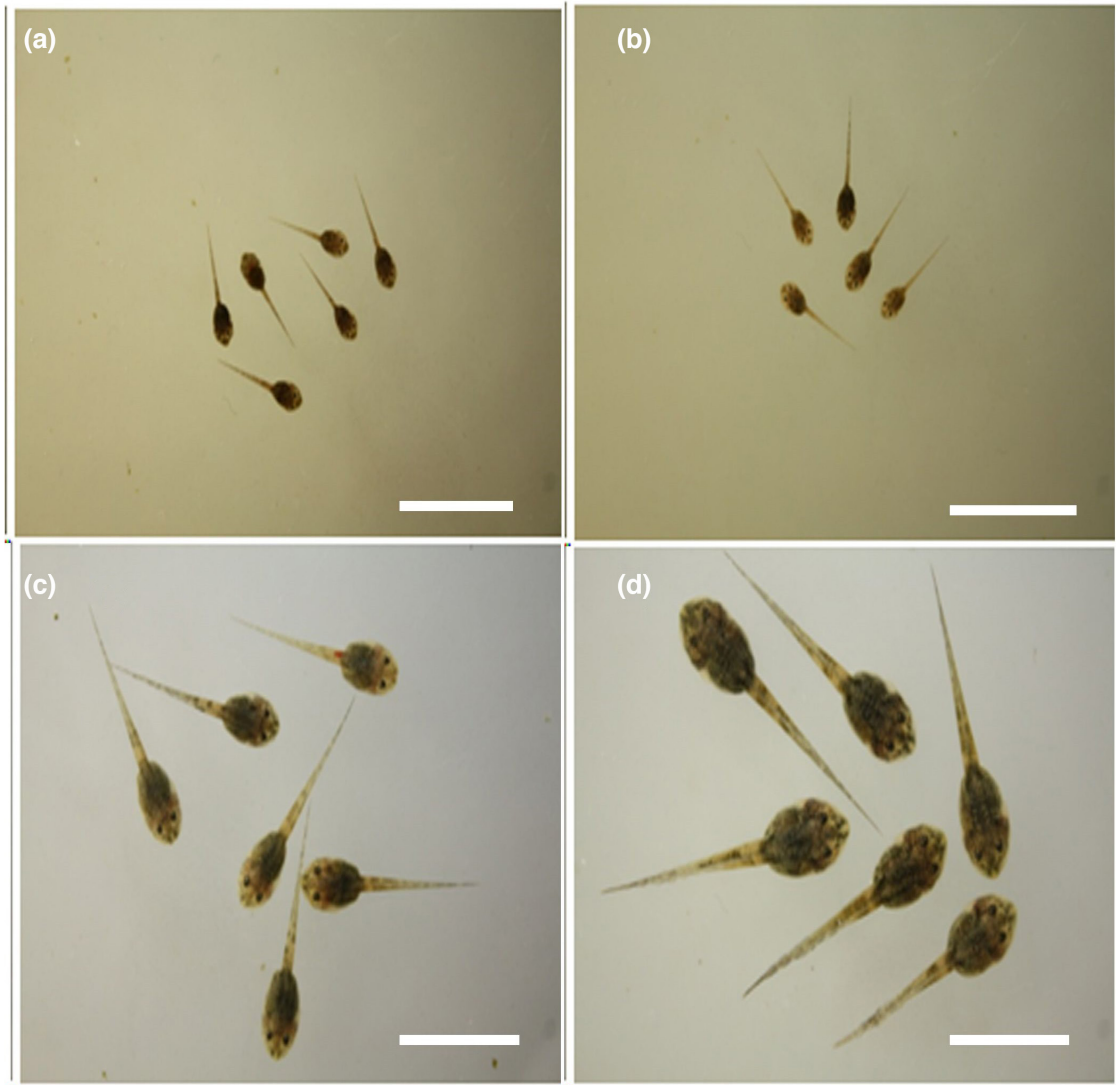
Twenty‐day‐old tadpoles of the hybrids and the controls among the *Fejervarya* from Asia. (a) Fkaw ♀ × Fisk ♂ (K), (b) Fkaw ♀ × Fisk ♂ (E), (c) Fkaw♀ × Fkaw ♂, (d) Fmul ♀ × Fkaw ♂. Scale bar = 10 mm.

**FIGURE 4:**
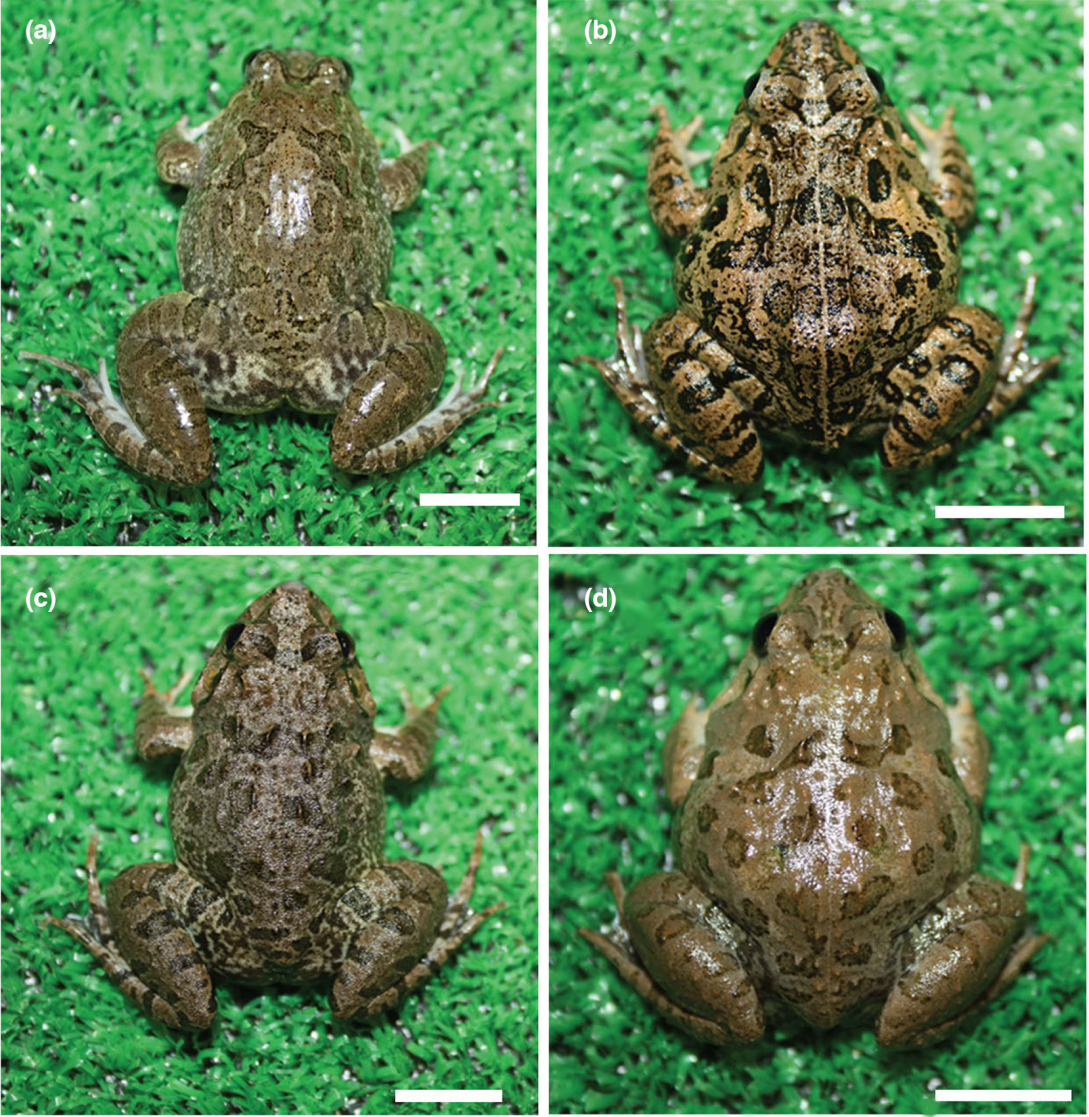
Dorsal views of 2‐year‐old control and hybrid frogs. (a) Fisk ♀ × Fisk ♂, (b) Fisk ♀ × Fver ♂, (c) Fver ♀ × Fisk ♂, (d) Fver ♀ × Fver ♂. Scale bar = 10 mm.

Reciprocal hybrids among *F*. *iskandari*, *F*. *verruculosa*, and *F*. sp. large type and those between *F*. *multistriata* and *F*. *kawamurai* were viable through metamorphosis. However, crosses between the *F*. *iskandari* group and *F*. *limnocharis* group were completely or partially inviable at the tadpole stage and those between Southeast Asian and South Asian *Fejervarya* groups were completely inviable at the embryonic stage (Table 2, Figures 3 and 4).

**TABLE 2:** Developmental capacity of hybrid and control offspring from crosses among *Fejervarya* species from lesser Sunda, Indonesia, and other Asian countries.

Date	Parents		No. of	No. of normally	No. of normal	No. of normally	No. of normally	No. of 23‐ to 30‐day‐old tadpoles (%)		No. of metamorphosed frogs (%)
	Female	Male	Eggs	Cleaved eggs (%)	Tail‐bud embryos (%)	Hatched tadpoles (%)	Feeding tadpoles (%)	Normal	Underdeveloped	
April 20.2010	Fisk(E)1	Fisk(E)	186	177 (95.2)	165 (88.7)	160 (86.0)	155 (83.3)	150 (80.6)	0 (0)	148 (79.5)
	Fisk(E)1	Fver(M)	345	329 (95.4)	132 (38.3)	130 (37.7)	128 (37.1)	121 (35.1)	0 (0)	115 (33.3)
	Fisk(E)1	Fkaw	190	175 (92.1)	165 (86.8)	140 (73.7)	138 (72.6)	115 (60.5)	0 (0)	114 (60.0)
	Fisk(E)1	Fsp.L	318	78 (24.5)	49 (15.4)	46 (14.5)	45 (14.2)	44 (13.8)	0 (0)	43 (13.5)
	Fisk(E)1	Fsp.S	295	140 (47.5)	136 (46.1)	130 (44.1)	2 (0.7)	0 (0)	—	—
	Fisk(E)2	Fisk(E)	139	135 (97.1)	130 (93.5)	125 (89.9)	121(87.1)	115 (82.7)	0 (0)	112 (80.5)
	Fisk(E)2	Fver(M)	195	167 (85.6)	75 (38.5)	74 (37.9)	74 (37.9)	73 (37.4)	0 (0)	73 (37.4)
	Fisk(E)2	Fkaw	128	97 (75.8)	39 (30.5)	38 (29.7)	37 (28.9)	36 (28.1)	0 (0)	35 (27.4)
	Fisk(E)2	Fsp.L	132	80 (60.6)	53 (40.2)	50 (37.9)	49 (37.1)	47 (35.6)	0 (0)	35 (26.5)
	Fisk(E)2	Fsp.S	200	190 (95.0)	55 (27.5)	40 (20.0)	0 (0)	—	—	—
	Fver(E)	Fisk(E)	115	84 (95.5)	76 (86.7)	75 (85.2)	72 (81.8)	70 (79.6)	0 (0)	69 (78.4)
	Fver(E)	Fver(M)	100	98 (98.0)	95 (95.0)	90 (90.0)	85 (85.0)	83 (83.0)	0 (0)	82 (82.0)
	Fver(E)	Fkaw	110	81 (73.6)	15 (13.6)	14 (12.7)	3 (2.7)	2 (1.8)	0 (0)	2 (1.8)
	Fver(E)	Fsp.L	27	10 (37.0)	7 (25.9)	6 (22.2)	6 (22.2)	5 (18.5)	0 (0)	5 (18.5)
	Fver(M)	Fisk(E)	206	203 (98.5)	165 (80.1)	162 (78.6)	155 (75.2)	150 (72.8)	0 (0)	141 (68.5)
	Fver(M)	Fver(M)	440	436 (99.1)	415 (94.4)	410 (93.2)	409 (92.3)	403 (91.6)	0 (0)	402 (91.4)
	Fver(M)	Fkaw	221	199 (90.1)	208 (94.1)	200 (90.5)	198 (89.6)	120 (54.3)	0 (0)	69 (31.2)
	Fver(M)	Fsp.L	287	155 (54.0)	72 (25.1)	62 (21.6)	60 (20.9)	57 (19.9)	0 (0)	48 (16.7)
	Fver(M)	Fsp.S	255	230 (90.2)	95 (37.3)	90 (35.3)	0 (0)	—	—	—
	Fsp.L	Fisk(E)	241	227 (94.2)	78 (32.4)	72 (29.9)	67 (27.8)	66 (27.4)	0 (0)	55 (22.8)
	Fsp.L	Fver(M)	400	350 (87.5)	24 (6.0)	23 (5.8)	18 (4.5)	16 (4.0)	0 (0)	15 (3.8)
	Fsp.L	Fsp.L	129	125 (96.8)	122 (94.5)	120 (93.0)	118 (91.4)	115 (89.2)	0 (0)	113 (87.6)
	Fsp.L	Fsp.S	162	130 (80.3)	0 (0)	—	—	—	—	—
May 10, 2012	Fkaw1	Fkaw2	168	119 (70.8)	107 (63.7)	107 (63.7)	97 (57.7)	90 (53.6)	0 (0)	85 (50.6)
	Fkaw1	Fisk(E)	99	7(7.0)	3 (3.0)	1 (1.0)	1 (1.0)	0 (0)	1 (1.0)	0 (0)
	Fkaw1	Fisk(K)	155	35 (22.5)	26 (16.8)	24 (15.5)	22 (14.2)	0 (0)	19 (12.3)	0 (0)
	Fkaw1	Fver(M)	178	9 (5.1)	1 (0.7)	1 (0.7)	0 (0)	—	—	—
	Fkaw1	Fsp.L	101	88 (87.1)	88 (87.3)	76 (75.3)	75 (74.3)	0 (0)	73 (72.3)	0 (0)
	Fkaw2	Fkaw2	202	94 (46.5)	83 (41.1)	76 (37.6)	72 (35.6)	70 (34.6)	0 (0)	68 (33.7)
	Fkaw2	Fisk(E)	108	9 (8.3)	7 (6.5)	5 (4.6)	5 (4.6)	0 (0)	5 (4.6)	0 (0)
	Fkaw2	Fisk(K)	156	143 (91.7)	129 (82.7)	91 (58.3)	80 (51.3)	0 (0)	69 (44.2)	0 (0)
	Fkaw2	Fver(M)	171	5 (2.9)	0 (0)	—	—	—	—	—
	Fkaw2	Fsp.L	161	152 (94.4)	144 (89.4)	139 (86.3)	135 (83.9)	0 (0)	129 (80.1)	0 (0)
	Fmul	Fkaw1	179	38 (21.2)	19 (10.6)	14 (7.8)	13 (7.3)	12 (6.7)	0 (0)	10 (5.6)
	Fmul	Fisk(K)	121	19 (15.7)	11 (9.0)	2 (1.7)	2 (1.7)	0 (0)	1 (0.8)	0(0)
	Fmul	Fver(M)	121	8 (6.6)	5 (4.1)	4 (3.3)	4 (3.3)	1 (0.8)	1 (0.8)	0(0)
	Fmul	Fsp.L	83	25 (31.1)	17 (20.5)	11 (13.3)	11 (13.3)	0 (0)	9 (10.8)	0(0)

### 3.2 Observations of the testes and spermatogenesis

**FIGURE 5:**
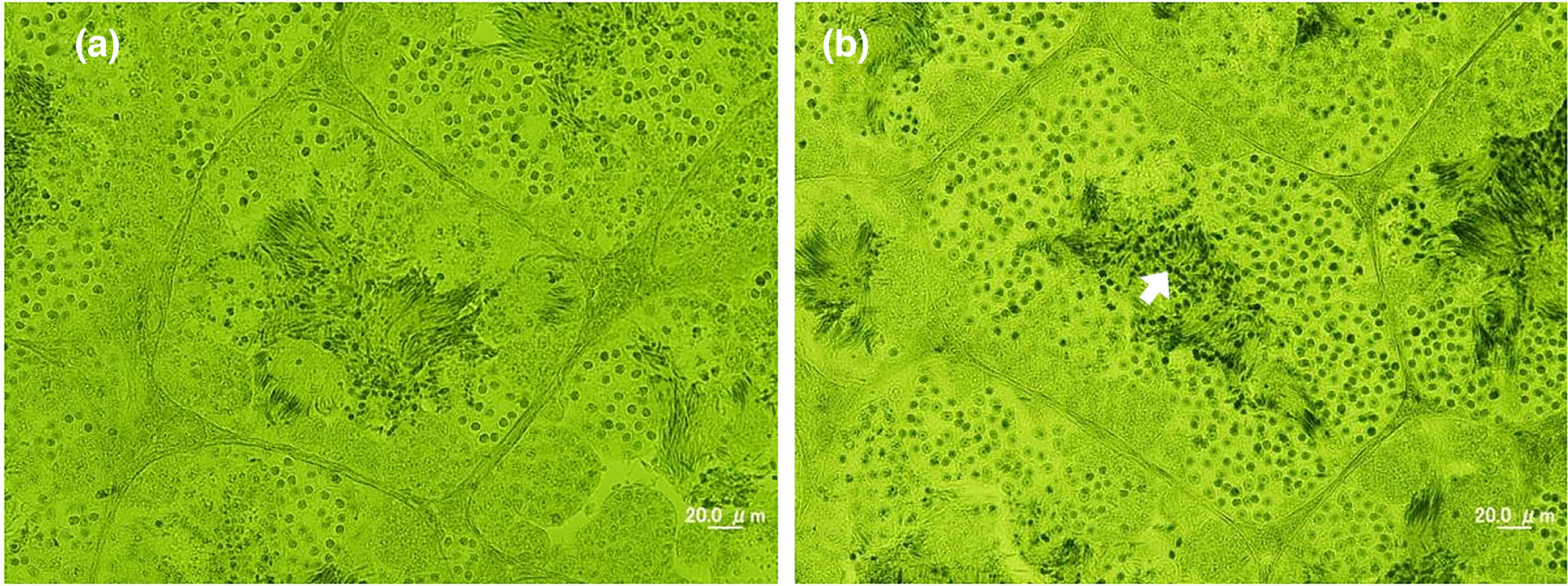
Cross‐sections of seminiferous tubules in the testes of the control and the hybrid. (a) Fver (M) ♀ × Fver (M) ♂, (b) Fver (M) ♀ × Fisk (E) ♂. Arrow shows pycnotic nuclei.

**FIGURE 6:**
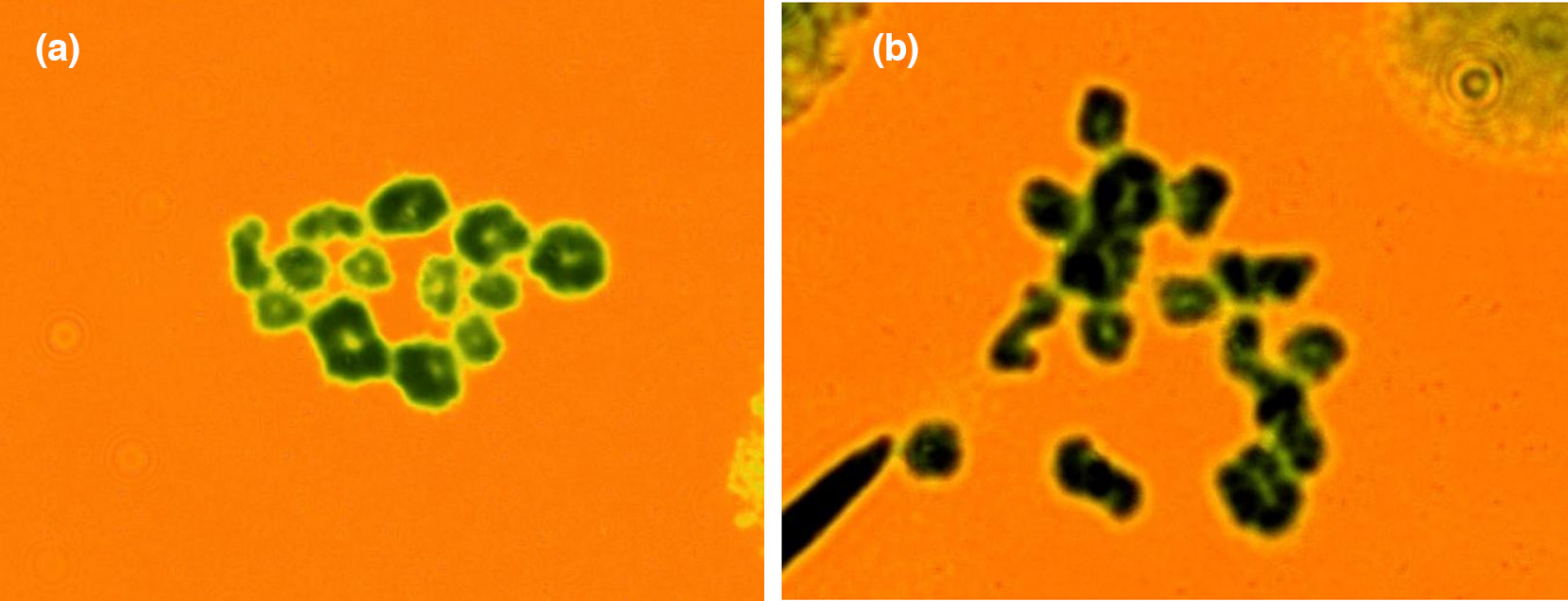
Spermatocytes at the first meiosis and chromosome complements in the control and the hybrid. (a) Fver (M) ♀ × Fver (M) ♂, (b) Fver (M) ♀ × Fisk (E) ♂. Control containing 13 bivalents, which are ring‐or rod‐shaped. Hybrid contained 2 univalents and 12 bivalents, which are ring‐or rod‐shaped.

To further clarify the relationships among these species, the inner structures of the testes from mature male hybrids between *F*. *verruculosa* and *F*. *iskandari* and the controls were quantified by histological analyses and observations of spermatogenesis (Figures 5 and 6). The inner arrangements of the testes of control males were completely normal; seminiferous tubules were filled with tight bundles of normal spermatozoa (Figure 5a). In contrast, the testes of the hybrids were slightly abnormal, with seminiferous tubules containing pycnotic nuclei in addition to normal bundles of spermatozoa (Figure 5b).

In controls, 26 meiotic spreads were interpreted from two males, all of which consisted of 13 bivalents and no univalents (Table 3, Figure 6a). In the hybrids, of the 41 meiotic spreads interpreted from four hybrid males, one (2.4%) consisted of 13 bivalents and no univalents, 16 (39.0%) consisted of 12 bivalents and 2 univalents, and 24 (58.5%) consisted of 11 bivalents and four univalents (Table 3, Figure 6b). The mean number of univalents per spermatocyte was 3.12 and the proportion of univalents among all chromosomes was 12.0% (Table 3). Ring‐shaped bivalents outnumbered rod‐shaped bivalents substantially in the controls, whereas relative frequencies of ring‐shaped bivalents decreased and rod‐shaped bivalents increased in the hybrids in both the large and small chromosomes (Table 4, Figure 6). In total, 104 (80.0%) and 146 (70.2%) large and small bivalent chromosomes were ring‐shaped in the controls, respectively, whereas 131 (68.2%) and 161 (49.7%) large and small bivalent chromosomes were ring‐shaped in the hybrids, respectively (Table 4). The mean number of bivalents per spermatocyte in the controls was 13.00, while that in the hybrids was 12.59 (Table 4). The mature reciprocal hybrids between *F*. *iskandari* and *F*. *verruculosa* from Indonesia, Lesser Sunda, showed some degree of abnormality in spermatogenesis (Figure 6).

**TABLE 3:** Number of meiotic spreads with differing numbers of univalents in male hybrids between *F*. *verruculosa* and *F*. *iskandari* and the control.

Parent		Number of meiosis	No. of univalents (%)					Mean no. of univalents per spermatocyte
Female	Male		0	2	4	8	10	
Fver(M)	Fver(M)	26	26 (100)	0	0	0	0	0
Fver(M)	Fisk(E)	41	1 (2.4)	16 (39.0)	24 (58.5)	0	0	3.12

**TABLE 4:** Number of ring‐ and rod‐shaped bivalents in male hybrids between *F*. *verruculosa* and *F*. *iskandari* and the control.

Parent		Total no. of bivalents	Large chromosome		Small chromosome		Total		Mean no. of bivalents per cell
Female	Male		Ring (%)	Rod (%)	Ring (%)	Rod (%)	Ring (%)	Rod (%)	
Fver(M)	Fver(M)	338	104 (80.0)	26 (20.0)	146 (70.2)	62 (29.8)	250 (74.0)	88 (26.0)	13.00
Fver(M)	Fisk(E)	516	131 (68.2)	61 (31.8)	161 (49.7)	163 (50.3)	292 (56.6)	224 (43.4)	12.59

### 3.3 mtDNA sequence data

**FIGURE 7:**
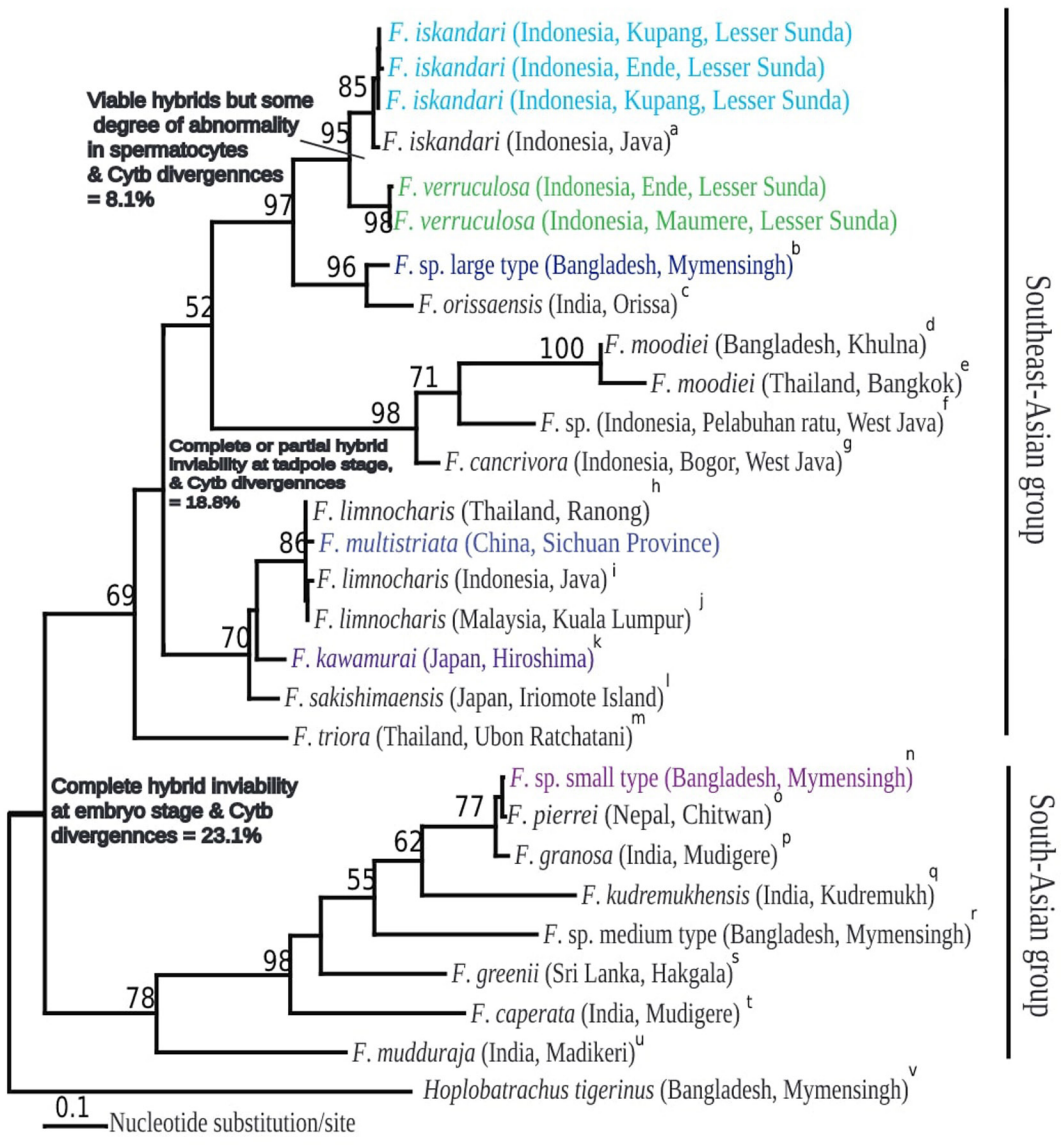
Phylogenetic tree constructed by the maximum likelihood (ML) method, based on nucleotide sequence of a 488‐bp segment of the mitochondrial *Cytb* gene. *Hoplobatrachus tigerinus* was used as an outgroup. Numbers on branches represent bootstrap support for ML inference. The scale bar represents 0.1 nucleotide substitutions per site.

Phylogenetic analyses based on *Cytb* sequences revealed that *F*. *iskandari* formed a sister clade with *F*. *verruculosa* from Lesser Sunda Indonesia with 8.1% sequence divergence. *Fejervarya multistriata* from China formed a clade with populations of *F*. *limnocharis* in Thailand, Malaysia, and Indonesian (Topotype), which showed sister relationships to *F*. *kawamurai* from Japan with 8.9% sequence divergence (Table 5, Figure 7). *Fejervarya* sp. small type from Bangladesh formed a clade with other South Asian members of the *Fejervarya* group and formed a sister clade with the Southeast Asian *Fejervarya* group with 23.7% sequence divergence (Figure 7).

**TABLE 5:** Nucleotide sequences of Cytb gene used for molecular analyses.

Species	Country	Location	Accession number	Source
*Fejervarya verruculosa*	Indonesia	Ende, Lesser Sunda	LC706527	Present study
		Maumere, Lesser Sunda	LC706529	Present study
*Fejervarya iskandari*	Indonesia	Ende, Lesser Sunda/Lowland	LC706533	Present study
		Kupang, Lesser Sunda	LC706535	Present study
		Kupang, Lesser Sunda	LC706536	Present study
		Java	^a^AB488813	Kotaki et al. (2010)
*Fejervarya multistriata*	China	Sichuan Province	LC706538	Present study
*Fejervarya* sp. Large type	Bangladesh	Mymensingh	^b^AB372046	Islam et al. (2008)
*Fejervarya orissaensis*	India	Orissa	^c^AB488842	Kotaki et al. (2010)
*Fejervarya moodiei*	Bangladesh	Khulna	^d^AB372069	Islam et al. (2008)
*Fejervarya moodiei*	Thailand	Bangkok	^e^AB444707	Kurniawan et al. (2010)
*Fejervarya* sp.	Indonesia	Pelabuhan ratu, West Java	^f^AB444709	Kurniawan et al. (2010)
*Fejervarya cancrivora*	Indonesia	Bogor, West Java	^g^AB444702	Kurniawan et al. (2010)
*Fejervarya limnocharis*	Thailand	Ranong	^h^AB488816	Kotaki et al. (2010)
*Fejervarya limnocharis*	Indonesia	Java	^i^AB488811	Kotaki et al. (2010)
*Fejervarya limnocharis*	Malaysia	Kuala Lumpur	^j^AB488815	Kotaki et al. (2010)
*Fejervarya kawamurai*	Japan	Hiroshima	^k^AB488832	Kotaki et al. (2010)
*Fejervarya sakishimaensis*	Japan	Iriomote Island	^l^AB488831	Kotaki et al. (2010)
*Fejervarya triora*	Thailand	Ubon Ratchathani	^m^AB4488820	Kotaki et al. (2010)
*Fejervarya* sp. Small type	Bangladesh	Mymensingh	^n^AB372058	Islam et al. (2008)
*Fejervarya pierrei*	Nepal	Chitwan	^o^AB488834	Kotaki et al. (2010)
*Fejervarya granosa*	India	Mudigere	^p^AB488844	Kotaki et al. (2010)
*Fejervarya* sp. Medium type	India	Kudremukh	^q^AB488849	Kotaki et al. (2010)
*Fejervarya kudremukhensis*	Bangladesh	Mymensingh	^r^AB372054	Islam et al. (2008)
*Fejervarya greenii*	Sri Lanka	Hakgala	^s^AB488838	Kotaki et al. (2010)
*Fejervarya caperata*	India	Mudigere	^t^AB488843	Kotaki et al. (2010)
*Fejervarya mudduraja*	India	Madikeri	^u^AB488845	Kotaki et al. (2010)
*Hoplobatrachus tigerinus*	Bangladesh	Mymensingh	^v^AP011543	Alam et al. (2010)

*Note*: Superscript letters indicate that *Cytb* data were taken from GenBank for use in constructing the tree as shown in Figure 7.

## 4 DISCUSSION

Speciation, the process by which new species evolve, is a fundamental issue in evolutionary biology and is closely connected to postmating isolation, genetics, and systematics. Mechanisms of postmating (reproductive) isolation are a useful tool to examine the accuracy of species delimitation. It is necessary to categorize sympatric or allopatric species groups (species with homoplasy/phenetic similarities) based on morphological, ecological, and genetic divergence. Wu and Hollocher (1998) reported that an interrelationship between genetic divergence and the degree of reproductive isolation might exist if the number of genes involved in reproductive isolation between taxa is large, with the continuous accumulation of mutations at these loci over time. Similar correlations have been found in *Drosophila* (Coyne & Orr, 1989), the salamander *Desmognathus ochrophaeus* (Tilley et al., 1990), and some anurans (Sasa et al., 1998). However, postmating isolation is not generally observed between all species, and analyses are limited by difficulty in crossing experiments as well as related costs and labor. Despite these barriers, we attempted to cross *Fejervarya* species from Lesser Sunda, Indonesia, with populations in other Asian countries to quantify the evolutionary relationships and postmating isolation among these frogs.

Reproductive isolation is indispensable for both the generation and preservation of flora and fauna (Dugas & Richards‐Zawacki, 2015). The breakdown of reproductive isolation can lead to gene exchange between species, resulting in the sterility or inviability of hybrid offspring (Arnold, 1997).

Futuyama (1986) claimed that reproductive isolation between two populations can be detected by direct observation, evaluations of mating properties, or examinations of the sterility and viability of hybrids produced in a controlled laboratory. Japanese evolutionary biologists started to examine reproductive isolation in amphibian in the 1980s. For example, Kawamura et al. (1980) evaluated the postmating isolation mechanism in Japanese, European, and American toads. Later, Kawamura et al. (1981, 1985) performed a series of artificial crossing experiments to determine the postmating isolation mechanism in brown frogs from various regions, including the United States, Europe, and Soviet Union. Sumida et al. (2003) evaluated the reproductive isolation mechanism and phylogenetic relationships among Palearctic and Oriental brown frogs, revealing the key roles of gametic isolation, hybrid inviability, and/or hybrid sterility and that viable interspecific hybrids were completely sterile males.

Both crossing experiments and mtDNA sequence analyses revealed that Southeast Asian frogs differed greatly from South Asian frogs. Crossing experiments demonstrated that the *F*. *iskandari* × *F*. *verruculosa* from Lesser Sunda, Indonesia, produces viable offspring, with some degree of abnormality in spermatocytes. *Cytb* sequence divergence between *F*. *iskandari* and *F*. *verruculosa* was 8.1%. Further, the *Cytb* sequence divergence was 18.8% between the *F*. *iskandari* group and *F*. *limnocharis* group, and crossing experiments showed complete or partial hybrid inviability at tadpole stage, with abnormal spermatogenesis in these two groups. Similar results were obtained by Sumida et al. (2007) and Djong et al. (2007).

Controlled crossing experiments are an essential tool in evolutionary genetics and have applications in population biology, ecology, and phylogenetics (Berger et al., 1994). Our crossing experiments demonstrated that Southeast Asian *Fejervarya* and South Asian *Fejervarya* groups are reproductively isolated due to complete hybrid inviability at the embryonic stage, with *Cytb* divergence of 23.1%. These results were in agreement with those of Sumida et al. (2007) and Djong et al. (2007). Postmating isolation between six species belonging to *Hoplobatrachus*, *Euphlyctis*, and *Fejervarya* was also quantified by Alam et al. (2012), with analyses of the degree of abnormality at the genus level, including the production of allotriploids by hybridization. Recently, South Asian frogs were transferred to the genus *Minervarya* based on observations by several herpetologists (Frost, 2021; Sanchez et al., 2018).

Histological quantification on the testes of hybrids between *F*. *iskandari* and *F*. *verruculosa* showed some abnormalities. For example, 2.4% consisted of 13 bivalents and 97.5% consisted of 2–4 univalents, with a mean number of univalents per spermatocyte of 3.12; frequencies of ring‐shaped and rod‐shaped bivalents were 56.6% and 43.3%, respectively. Callan and Spurway. (1951) reported 0.9–4.3 (mean 2.44) univalents per spermatocyte in hybrids of two European newts, *Triturus cristatus carnifex* (=*T*. *carnifex*) and *Triturus cristatus karelinii* (=*T*. *karelinii*). In anurans, the mature reciprocal hybrids between *F. iskandari* and *F. verruculosa* from Indonesia, Lesser Sunda, showed some degree of abnormality in spermatogenesis. Hasan et al. (2017) also revealed that in reciprocal hybrids between *H*. *tigerinus* and *H*. *litoralis*, the mean univalents per spermatocyte were 0.01 and 0.17, and the frequencies of rod‐shaped bivalents were 23.7% and 25.5%. These results showed that *H. litoralis* and *H*. *tigerinus* were not isolated by hybrid inviability or by hybrid sterility; however, they showed a slight divergence, as evidenced by somewhat abnormal spermatogenesis.

## 5 CONCLUSION

Reciprocal hybrids between *F. iskandari* and *F. verruculosa* generated in the laboratory were viable. Complete or partial hybrid inviability was observed between *F. limnocharis* and *F. iskandari* groups; however, hybrid inviability was complete between Southeast Asian and South Asian populations of *Fejervarya* frogs at the embryonic stage. Phylogenetic analyses based on *Cytb* sequences revealed that *F. iskandari* formed a sister clade with *F. verruculosa* from Lesser Sunda, Indonesia, with 8.1% sequence divergence. From an evolutionary perspective, *F. kawamurai* from Japan is closely related to Southeast Asian populations in the *F. limnocharis* group and distantly related to the *F. iskandari* group. The sister relationships between Southeast Asian and South Asian members of *Fejervarya* and substantial genetic divergence (*Cytb* = 23.1%) were observed. This study proved that the degree of postmating isolation imitates molecular phylogenetic affinities. The two species *F. iskandari* and *F. verruculosa* from Indonesia (Lesser Sunda) were not separated by gametic isolation, hybrid inviability, or hybrid sterility. However, detailed observations showed that hybrid males show some abnormalities in spermatogenesis. Therefore, the two species are isolated by abnormal spermatogenesis and show some degree of genetic divergence. Sumida et al. (2007) claimed that phylogenetic relationships among taxa are closely related to the degree of reproductive isolation, consistent with our results indicating that relationships in the phylogenetic tree are consistent with results of crossing experiments. Further sampling is necessary from the Indonesian Archipelago (Sundaland, Wallacea, and the Australian region) to elucidate all aspects of speciation in the genus *Fejervarya* in the area.

## AUTHOR CONTRIBUTIONS

**Mahmudul Hasan:** Conceptualization (lead); data curation (lead); formal analysis (equal); methodology (equal); writing – original draft (lead); writing – review and editing (lead). **Nia Kurniawan:** Conceptualization (equal); data curation (equal); methodology (equal); validation (supporting). **Aris Soewondo:** Data curation (equal); methodology (equal). **Wilmientje Marlene Mesang Nalley:** Investigation (supporting); methodology (equal). **Masafumi Matsui:** Conceptualization (equal); investigation (equal); methodology (equal). **Takeshi Igawa:** Formal analysis (equal); methodology (equal). **Masayuki Sumida:** Conceptualization (equal); data curation (equal); methodology (equal); project administration (lead); supervision (lead).

## CONFLICT OF INTEREST

The authors have declared that no competing interests exist.

## Data Availability

The data related to this article can be accessed here: https://doi.org/10.5061/dryad.np5hqbzxf.
